# The impact of heat on mortality and morbidity in the Greater Metropolitan Sydney Region: a case crossover analysis

**DOI:** 10.1186/1476-069X-12-98

**Published:** 2013-11-15

**Authors:** Leigh Ann Wilson, Geoffrey Gerard Morgan, Ivan Charles Hanigan, Fay H Johnston, Hisham Abu-Rayya, Richard Broome, Clive Gaskin, Bin Jalaludin

**Affiliations:** 1 Faculty of Health Science, University of Sydney, Sydney, Australia; 2 School of Science and Health, University of Western Sydney, Sydney, Australia; 3 University Centre for Rural Health – North Coast, University of Sydney, Sydney, Australia; 4 North Coast Public Health Unit, Mid North Coast Local Health District, New South Wales, Australia; 5 National Centre for Epidemiology and Population Health, Australian National University, Acton, Australia; 6 Menzies Research Institute, University of Tasmania, Hobart, Australia; 7 Centre for Epidemiology and Research, NSW Health, Sydney, Australia; 8 Health Protection New South Wales, Sydney, Australia; 9 NSW Cancer Institute, Sydney, Australia; 10 School of Public Health and Community Medicine, University of New South Wales, Sydney, Australia; 11 Centre for Research, Evidence Management and Surveillance, South Western Sydney Local Health District, Sydney, Australia

**Keywords:** Climate change, Heat-wave, Heat-related illness, Heat threshold, Case-crossover design

## Abstract

**Background:**

This study examined the association between unusually high temperature and daily mortality (1997–2007) and hospital admissions (1997–2010) in the Sydney Greater Metropolitan Region (GMR) to assist in the development of targeted health programs designed to minimise the public health impact of extreme heat.

**Methods:**

Sydney GMR was categorized into five climate zones. Heat-events were defined as severe or extreme. Using a time-stratified case-crossover design with a conditional logistic regression model we adjusted for influenza epidemics, public holidays, and climate zone. Odds ratios (OR) and 95% confidence intervals were estimated for associations between daily mortality and hospital admissions with heat-event days compared to non-heat event days for single and three day heat-events.

**Results:**

All-cause mortality overall had similar magnitude associations with single day and three day extreme and severe events as did all cardiovascular mortality. Respiratory mortality was associated with single day and three day severe events (95^th^percentile, lag0: OR = 1.14; 95%CI: 1.04 to 1.24). Diabetes mortality had similar magnitude associations with single day and three day severe events (95^th^percentile, lag0: OR = 1.22; 95%CI: 1.03 to 1.46) but was not associated with extreme events. Hospital admissions for heat related injuries, dehydration, and other fluid disorders were associated with single day and three day extreme and severe events. Contrary to our findings for mortality, we found inconsistent and sometimes inverse associations for extreme and severe events with cardiovascular disease and respiratory disease hospital admissions. Controlling for air pollutants did not influence the mortality associations but reduced the magnitude of the associations with hospital admissions particularly for ozone and respiratory disease.

**Conclusions:**

Single and three day events of unusually high temperatures in Sydney are associated with similar magnitude increases in mortality and hospital admissions. The trend towards an inverse association between cardio-vascular admissions and heat-events and the strong positive association between cardio-vascular mortality and heat-events suggests these events may lead to a rapid deterioration in persons with existing cardio-vascular disease resulting in death. To reduce the adverse effects of high temperatures over multiple days, and less extreme but more frequent temperatures over single days, targeted public health messages are critical.

## Introduction

Extreme heat events are known to contribute to an increase in mortality and morbidity, particularly in vulnerable populations [1,2]. Early Heat Health Warning Systems (HHWS) have been shown to reduce morbidity and mortality during heat-waves (periods of unusually high temperature) [3-6]. HHWS rely on a number of components including timely and reliable meteorological data, an understanding of who is at increased health risk due to heat-waves and effective public health risk communication [7]. There is mounting evidence that heat-waves are increasing in frequency, duration and intensity as part of climate change [1]. Studies indicate that populations acclimatise to their regional climate and that a population’s response to a heat-wave is influenced by this acclimatisation [8]. Understanding the impact of climate on population health is the key to reducing the burden of disease due to climate change in Australia [9].

Much of the research linking heat-waves to excess mortality and morbidity has been conducted in the regions of the northern hemisphere where heat-waves are rare events [10-12], although some northern hemisphere locations do experience heatwaves on a regular basis [13-15]. Although heat-related mortality [9,16-19] and morbidity [9,18-21] studies have been conducted in Australia, study methods and the population groups assessed have varied considerably. This study builds on recent research conducted by Khalaj and colleagues [9] in the Sydney Greater Metropolitan Region (GMR) which used a case-only design to identify underlying conditions that modify the health risk associated with heat-waves. Khalaj and colleagues [9] found that people admitted to hospital with underlying mental and behavioural disorders, diseases of the nervous and circulatory system, diseases of the respiratory system and renal disease are more susceptible to extreme heat events. The case-only study design used by Khalaj is useful in determining which groups of patients are more susceptible to extreme heat events but cannot be used to provide quantitative estimates of the overall increase in deaths or the demand for health services during such events.

This study aims to quantify the association between unusual heat events and mortality as well as morbidity (hospital admission) in metropolitan New South Wales (NSW), Australia and inform the development of a state-wide HHWS. In addition, this study will help determine the extent to which northern hemisphere studies are generalisable to an Australian setting where the temperature threshold of unusual heat events is substantially higher than in the northern hemisphere [9]. In Australia there is lack of consensus on the definition of unusual heat events with the Australian Bureau of Meteorology defining a heat-wave as a period of more than a few days in a row of above average temperatures for a given location [22].

## Methods

### Data

Mortality data for all deaths of NSW residents between 1st July 1997 and 31st December 2007 (inclusive) were obtained from the Australian Bureau of Statistics (ABS) [23]. Data on all hospital admissions of NSW residents from 1st July 1997 to 30th June 2010 inclusive were obtained from the NSW Department of Health [24]. Meteorological data on temperature (daily minimum, maximum, mean), humidity (daily minimum, maximum, mean) and wind speed (at 9 am and 3 pm) were obtained from the Australian Bureau of Meteorology (BoM) for all monitoring stations in the Sydney Greater Metropolitan Area (GMR) from 1997 to 2010.

### Climate zones

**Figure 1:**
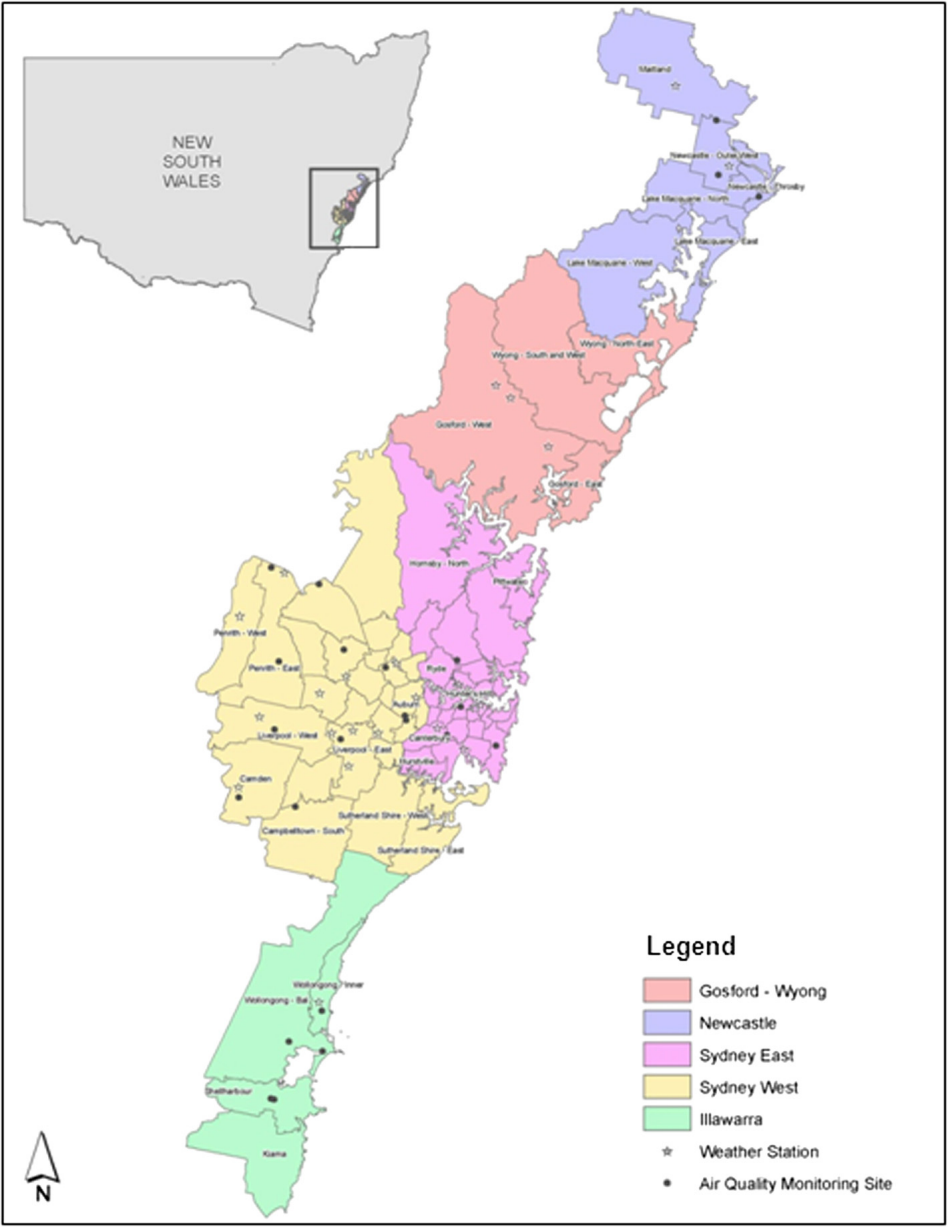
Map of study region including temperature zones and weather and air pollution monitoring stations.

Our analysis focused on the (southern hemisphere) spring and summer months of September to February. We included spring months because early heat-waves can occur during this time and poor acclimatisation to warm weather following the winter months has the potential to increase the likelihood of heat-related illness [8]. Sydney experiences cooler nights in the inland (Sydney west) zone, and cooler days in the coastal (Sydney east) zone. Previous work by Khalaj et al. [9] used data from all Sydney weather stations to define two Sydney climate zones (Sydney east, Sydney west) by maximising the ratio of between-region to within-region temperature variance. In this study Statistical Local Area (SLA) of residence was used to assign mortality and hospital admissions data to each of the five climate zones of Sydney east, Sydney west, Gosford/Wyong, Newcastle, Illawarra [25]. Daily weather data were obtained for all BoM stations within 50 km of the population-weighted centre of each SLA [26]. The daily average was calculated as the inverse distance weighted average of all validated observations on each day. The distance weighted averages from the population centres were used because we assumed these estimates more closely relate to the experience of the population, rather than that at airports or other locations [27]. The population centre was calculated using data from the 2006 Australian Census [28]. Figure 1 illustrates the study region including the temperature zones, weather and air pollution monitoring stations.

### Health outcome measures

Hospital admission and mortality data based upon study subjects’ usual place of residence were mapped to SLA 2006 boundaries. While our literature review identified many variations in disease categorisations for both mortality and hospital admissions in previous studies, these variations were generally minor and concerned relatively infrequent diagnoses.

Mortality was grouped into categories based on the underlying cause of death (i.e. the disease or injury which initiated the train of events leading directly to death). Categories used were based on International Classification of Diseases – 10 (ICD-10) coding informed by previous research (Additional file 1). Hospital admissions were analysed in similar groups based on ICD-10 coding (Additional file 1).

### Definitions of heat

We defined heat-waves using two heat metrics: daily apparent maximum air temperature (TAppMax) [29] and daily maximum temperature (Tmax). Apparent temperature is a measure of relative discomfort due to combined heat and high humidity. This measure was developed by Steadman [30,31] on the basis of physiologic studies on evaporative skin cooling and can be calculated as a combination of air temperature and dew point. The occurrence of negative dew point values in Australian data makes the use of the commonly used Kalkstein and Valimont [32] apparent temperature formula invalid as it involves taking the square of dewpoint, which introduces a sign error on days of low humidity. In this study we used the Steadman [33] formula. Daily maximum temperature is the highest temperature reached in a given location over a given 24 hour period from midnight to midnight.

We defined single day severe heat events as days where the temperature metric exceeds the 95^th^percentile of the monthly distribution for the study period in that zone. Similarly, three day severe heat events are defined as days when the three day moving average (lags 0–2) exceeds the 95^th^percentile. We then defined single day extreme events as days when the temperature metric exceeds the 99^th^percentile of the monthly distribution for the study period in that zone, and three day extreme events (or heat-waves), as days when the three day moving average (lags 0–2) exceeds the 99^th^percentile. The association between mortality and hospital admissions with severe events provides information on the health effects associated with relatively regular heat events (on average nine events during spring and summer each year), while the association with extreme events provides information on the health effects that occur during less frequent heat events (on average two events per year).

Additional sensitivity analyses which assessed four alternative heat event definitions for associations with mortality from 1) all causes, 2) all cardiovascular disease and 3) all respiratory disease were conducted. These alternative heat-wave definitions were proposed by the Australian Bureau of Meteorology and the NSW Ministry of Health to capture the integrated experience of high temperatures over multiple days. The Excess Heat Factor (EHF) index proposed by Nairn et al. [34] calculates the difference between the 3 day moving average (lags 0–2) of daily average temperature and the prior 30 day moving average (lags 3–32). This is then amplified by the difference of the three day average and the 95th percentile of daily average temperatures. Days with an EHF > 1 are defined as “heat-wave” days (EHF severe). As EHF > 1 occurs relatively frequently we also defined “extreme heat-wave” days as EHF > 80 percentile value (EHF extreme). The third definition which is NSW specific (NSW1) [35] identifies a heat wave as two consecutive days greater than the 95th percentile of maximum temperature for a given month. The fourth definition, also NSW specific (NSW2), proposes that a heat wave is any three day period with greater than the 90th percentile of maximum temperatures for that month in addition to a moving average of greater than 27°C maximum temperature for the 3 day period.

### Statistical analyses

We used a time-stratified case-crossover design, in which each case is their own control [36]. Comparisons were made between the ‘event’ day (the day the case was admitted to hospital or died) and several referent ‘non-event’ days, with measured and unmeasured potential confounding factors such as age and smoking status controlled by design. The referent days were selected from the same month and year and matched by day of week to the health outcome. This time-stratified method of selecting comparison days ensures unbiased conditional logistic regression estimates and avoids bias resulting from time trends in examination of the environmental exposures [37]. We used conditional logistic regression models to calculate odds ratios (OR) and 95% confidence intervals (CI) for mortality and hospital admission on *extreme* heat days (lag0, lag1, lag2, lag3, lag 0–2 days) compared to other days, adjusted for potential confounders and stratified by zone (ie: including an indicator for zone). The covariates were chosen a priori based on our literature review and were included in all models.

To control for influenza in the model we included a dummy variable for daily counts of influenza admissions (ICD 10: J09 – J18) greater than the 90^th^percentile of the Sydney GMR distribution. Single days within an influenza event lasting several days/weeks may dip below the 90^th^percentile and we included these days as influenza event days. In addition, an indicator variable was added to the model to account for public holidays. School holidays were also included as an indicator variable when hospital admissions for asthma was the outcome as this group includes a substantial proportion of children. Table 1 summarises the covariates included in the statistical model (Table 1).

**Table 1:** Covariates included in statistical models

**Exposure variable**	**Definition**
TempMax (TMax) (°C)	Same day temperature max - lag0, lag1, lag2, lag3, av (lags0-2)
DewPointMax (°C)	Same day dew point temperature max– lag0
Flu Epidemic(0,1)	Binary variable coded as 1 if influenza hospital admission rates were < 90th percentile and otherwise 0
TempAppMax (TAppMax)	Same day Apparent Temperature Max - lag0, lag1, lag2, lag3, av(lags0-3)
School holiday	Binary variable coded as 1 if school holidays and otherwise 0. Only included in childhood asthma models.
Public holiday	Binary variable coded as 1 if public holiday and otherwise 0.
Region	Dummy variable/s for regions

Air pollution concentrations may also be elevated during extreme and severe heat events and so potential confounding by air pollution was assessed by conducting sensitivity analyses that included air pollutants (PM_10_ (24 hour average), PM_2.5_ (24 hour average), NO_2_ (1 hour maximum) and O_3_ (1 hour maximum)) separately in the models. Data on air pollutants were obtained from the NSW Office of Environment and Heritage. Daily region-wide average air pollution concentrations were calculated using daily data from all available monitoring stations in each of the five zones (monitoring stations: Sydney east, n = 4; Sydney west, n = 7; Gosford/Wyong, n = 0; Newcastle, n = 3; Illawarra, n = 3). Gosford/Wyong did not have any monitoring stations so those from the neighbouring city of Newcastle were used.

## Results

**Figure 2:**
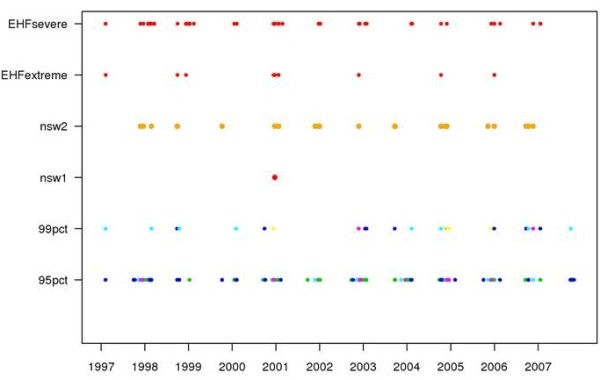
Heat event days as defined by the 99th percentile and 95th percentile for Sydney East for the period 1997 to 2007, as well as the four alternative heat wave definitions.

Temperature distributions varied across the five climate zones studied and summary statistics including temperature thresholds are presented in Table 2. The monthly 95^th^percentile thresholds in Sydney east ranged from 27.6°C in September to 32.8°C in January, while in Sydney west they ranged from 28.5°C in September to 35.4°C in January. Figure 2 shows heat event days as defined by the 99^th^percentile and 95^th^percentile for Sydney east for the period 1997 to 2007, as well as the four alternative heat wave definitions.

**Table 2:** Descriptive temperature statistics by region over the study periods (1997 to 2010)

**Daily temperature (°C)**	**Min**	**Max**	**Mean**	**95th percentile range (min – max)**	**99th percentile range (min – max)**
Sydney East	11.3	43.0	21.9	27.6 – 32.8	30.5 – 36.7
Sydney West	10.8	43.3	22.4	28.2 – 35.4	31.5 – 41.4
Newcastle	9.4	42.0	21.6	26.8 – 32.5	29.9 – 36.4
Gosford	11.0	43.3	20.8	26.3 – 29.6	28.6 – 33.9
Illawarra	10.9	41.8	22.1	27.9 – 33.6	31.3 – 37.2

As the focus of this study is to inform the development of a HHWS, comparisons were made between mortality and hospital admissions associated with rare occurrences of single day and three day extreme heat events, and those of the more frequent single day and three day severe heat events. Results from the statistical model (without controlling for air pollutants) are reported. Including air pollutants in the model had little influence on the mortality results but did influence some results for hospital admissions and these are highlighted in the text.

We found broadly similar associations between mortality and hospital admissions with single day events as well as three day events (lag 0 – 2). The largest single day lag was generally same day (lag0) although this did vary for hospital admissions. The results were also similar for extreme heat events and severe heat events defined using daily maximum temperature and daily maximum apparent temperature. The magnitude of the associations between the rare extreme events (99^th^percentile definition) and the more frequent severe events (95^th^percentile definition) was also generally similar. The magnitude of the associations was generally similar for all-age and 65 + years. The asthma hospital admissions results are reported for childhood asthma (1–14 years) and adult asthma (15–64 years) rather than all age asthma hospital admissions due to the different epidemiology of asthma in children and adults. The associations for mortality and hospital admissions with severe heat events (95^th^percentile threshold) and extreme heat events (99^th^percentile threshold) defined using maximum daily temperature are summarized in Table 3 and Table 4 respectively. The results highlighted in the text below are generally for single day severe events as the results for multiple day severe events and single and multiple day extreme events were usually similar. Results are presented in parentheses as percentile threshold, lag: (OR: 95% LCI – 95% UCI).

**Table 3:** Daily mortality statistics by cause of death in greater metropolitan Sydney regions 28th Feb 1997 – 2007

**Cause of death (ICD-10)**	**Greater Metropolitan Sydney Region**		
	**Daily deaths by cause**		
	**Min (Range)**	**Max (Range)**	**Median (Range)**
All causes (ICD-10 A00 – R99)	0 – 16	14 – 62	4 – 33
Cardiac arrest (ICD-10 I46)	0 – 0	1 – 2	0 – 0
Ischaemic heart diseases (ICD-10 I20 – 125.9)	0 – 0	5 – 16	1 – 6
CVD (ICD-10 I00 – 199.9)	0 – 3	8 – 34	0 – 3
Cardiovascular disease (ICD-10 I01 – I51.9)	0 – 1	6 – 24	1 – 9
Atrial fibrillation (ICD-10 I48 – I48.9)	0 – 0	2 – 3	0 – 0
Myocardial infarct (ICD-10 I21 – I22.9)	0 – 0	5 – 11	0 – 3
Congestive heart failure (ICD-10 I50 – I50.9)	0 – 0	2 – 5	0 – 0
Cerebrovascular disease (ICD-10 I60 – I69.9)	0 – 0	4 – 15	0 – 4
Stroke (ICD-10 I64 – I64.9)	0 – 0	3 – 6	0 – 1
Respiratory disorders (ICD-10 J00 – J99.9)	0 – 0	3 – 6	0 – 3
COPD (ICD-10 J40 – J44.9 or ICD-10 J47 – J47.9)	0 – 0	3 – 6	0 – 1
Pneumonia (ICD-10 J12 – J18.9)	0 – 0	2 – 6	0 – 0
Mental disorders (ICD-F00 – F99.9)	0 – 0	2 – 8	0 – 1
Mental (substance abuse) (ICD-F10 – F19.9)	0 – 0	2 – 4	0 – 0
Dementia (ICD-10 F00 – F03.9)	0 – 0	2 – 8	0 – 0
Diabetes mellitus (ICD-E10 – E14.9)	0 – 0	2 – 4	0 – 0
Schizophrenia (ICD-F20 – F29)	0 – 0	2 – 4	0 – 0
Suicide (ICD-10 ×60 – ×84.9 or Y870)	0 – 0	2 – 4	0 – 0
Dehydration (ICD-10 E86 – E86.9)	0 – 0	1 – 2	0 – 0
Disorders of fluid balance (ICD-10 E86 – E86.9)	0 – 0	1 – 2	0 – 0
Renal failure (ICD-10 N17 – N19.9)	0 – 0	2 – 4	0 – 0

**Table 4:** Daily hospital admission statistics by condition in greater metropolitan Sydney region

**Condition (ICD-10)**	**Greater Metropolitan Sydney Region**		
	**Daily admissions by cause**		
	**Min (Range)**	**Max (Range)**	**Median (Range)**
All causes (ICD-10 A00 – R99)	24 – 206	113 – 515	63 – 333
Cardiac arrest (ICD-10 I46)	0 – 0	2 – 4	0 – 0
Ischaemic heart diseases (ICD-10 I20 – 125.9)	0 – 4	15 – 37	4 – 17
CVD (ICD-10 I00 – 199.9)	1 – 20	27 – 90	10 – 52
Cardiovascular disease (ICD-10 I01 – I51.9)	0 – 13	23 – 68	7 – 38
Cerebrovascular disease (ICD-10 I60 – I69.9)	0 – 0	7 – 21	1 – 8
Respiratory disorders (ICD-10 J00 – J99.9)	0 – 14	22 – 93	7 – 43
COPD (ICD-10 J40 – J44.9 or ICD-10 J47 – J47.9)	0 – 1	11 – 35	2 – 9
Pneumonia (ICD-10 J12 – J18.9)	0 – 2	8 – 31	1 – 10
Asthma All Ages	0 – 2	8 – 48	1 – 8.5
Mental disorders (ICD-F00 – F99.9)	0 – 9	15 – 56	4 – 25
Mental (substance abuse) (ICD-F10 – F19.9)	0 – 0	6 – 26	1 – 7
Dementia (ICD-10 F00 – F03.9)	0 – 0	3 – 7	0 – 1
Schizophrenia (ICD-F20 – F29)	0 – 0	6 – 20	0 – 6
Renal failure (ICD-10 N17 – N19.9)	0 – 0	4 – 8	0 – 2
Urolithiasis (ICD-10 N20– N23)	0 – 0	6 – 17	0 – 4.5
Dehydration (ICD-10 E86 – E86.9)	0 – 0	4 – 7	0 – 1
Disorders of fluid balance (ICD-10 E87 – E87.8)	0 – 0	4 – 7	0 – 1
Diabetes mellitus (ICD-E10 – E14.9)	0 – 0	6 – 17	1 – 5
Heat related injuries (ICD-10 T67 – T67.9)	0 – 0	2 – 8	0 – 0
Neurotic /Somatic/Stress (ICD-10 F40 – F48)	0 – 0	5 – 23	1 – 3

All-cause mortality had similar magnitude associations with single day and three day extreme and severe events (95^th^percentile lag0 (1.06: 1.03 – 1.09)). All cause hospital admissions also showed similar small magnitude associations with three day and single day extreme and severe events (95^th^percentile, lag0 (1.02: 1.01 – 1.03)) although only the association with three day extreme events remained when ozone was included in the model (Table 5).

**Table 5:** Odds ratios of mortality associated with severe and extreme heat (95th/99th percentile) in Sydney GMR

**Deaths**		**Single day**		**3 day event**	
		**(Lag0)**		**(Lag0-2)**	
		**OR (95%CI)**		**OR (95%CI)**	
		**95th percentile**	**99th percentile**	**95th percentile**	**99th percentile**
All cause	All Age	*1.06 (1.03 - 1.09)*	*1.07 (1.02 – 1.13)*	*1.06 (1.04 - 1.09)*	*1.09 (1.03 – 1.15)*
	65+	*1.05 (1.02 - 1.08)*	1.05 (0.99 - 1.12)	*1.05 (1.02 - 1.08)*	*1.09 (1.03 - 1.15)*
All cardiovascular	All Age	*1.06 (1.01 - 1.12)*	*1.07 (0.97 - 1.18)*	*1.11 (1.06 - 1.17)*	*1.17 (1.06 - 1.29)*
	65+	*1.06 (1.01 - 1.12)*	1.09 (0.98 - 1.20)	*1.11 (1.05 - 1.17)*	*1.20 (1.08 - 1.32)*
IHD	All Age	*1.07 ( 1.01 - 1.14)*	*1.12 (1.01 - 1.26)*	*1.12 (1.06 - 1.18)*	*1.15 ( 1.03 - 1.29)*
	65+	*1.06 (1.00 - 1.13)*	*1.13 (1.01 - 1.27)*	*1.10 (1.04 - 1.18)*	*1.17 (1.04 - 1.32)*
All respiratory	All Age	*1.14 (1.04 - 1.24)*	1.08 (0.91 - 1.29)	1.08 (0.99 - 1.18)	0.99 (0.83 - 1.19)
	65+	*1.13 (1.03 - 1.23)*	1.09 (0.91 - 1.30)	1.07 (0.97 - 1.17)	0.97 (0.80 - 1.18)
Diabetes	All Age	*1.22 (1.03 - 1.46)*	1.13 (0.81 - 1.58)	1.16 (0.97 - 1.39)	0.88 (0.59 - 1.32)
	65+	*1.26 (1.05 - 1.51)*	1.20 (0.83 - 1.70)	1.14 (0.94 - 1.39)	0.92 (0.60 - 1.40)
Renal failure	All Age	1.18 (0.95 - 1.45)	1.14 (0.75 - 1.73)	1.09 (0.88 - 1.35)	0.89 (0.54 - 1.46)
	65+	1.17 (0.94 - 1.45)	1.08 (0.69 - 1.67)	1.05 (0.83 - 1.31)	0.90 (0.54 - 1.50)
Mental disorders	All Age	1.10 (0.95 - 1.27)	0.88 (0.64 - 1.20)	1.04 (0.90 - 1.21)	0.93 (0.68 - 1.28)
	65+	1.07 (0.91 - 1.27)	0.82 (0.58 - 1.16)	0.98 (0.83 - 1.16)	0.81 (0.56 - 1.66)
Disorders of fluid balance	All Age	1.10 (0.46 - 2.63)	1.98 (0.49 - 8.06)	*2.53 (1.18 - 5.41)*	*5.63 (1.62 - 19.56)*
	65+	1.02 (0.39 - 2.60)	2.37 (0.55 - 10.17)	*2.37 (1.00 - 5.61)*	0.66 (0.23 - 1.97)

Mortality from disorders of fluid, electrolyte and acid–base balance had large magnitude associations for single day (lag 1 day) and three day extreme and severe events (95^th^percentile, lag1 (4.94:2.16 – 11.29)). However, mortality from dehydration was only weakly associated with single day (lag 1 day) and three day severe events (95^th^percentile, lag1 (1.54: 0.93 – 2.53)) and not with extreme events. As expected we found large magnitude associations with hospital admissions for heat related injury and illness with single day and three day severe and extreme events (95^th^percentile, lag0 (12.05: 8.91 – 16.30)) although controlling for air pollution approximately halved the magnitude of these associations. Three day and single day extreme and severe events showed similar magnitude associations with hospital admissions for dehydration (95^th^percentile, lag0 (1.77: 1.60 – 1.97)) and admissions for disorders of fluid, electrolyte and acid base balance (95^th^percentile, lag0 (1.27:1.14 – 1.42)), while controlling for ozone marginally reduced the magnitude of these associations.

Cardiovascular mortality was associated with single day and three day extreme and severe events and the magnitude of these associations were larger for multiple day events compared to single day events (95^th^percentile, lag0 (1.06: 1.01 – 1.12)) and these results were reflected in ischaemic heart disease mortality (95^th^percentile, lag0 (1.07: 1.01 – 1.14)). Cerebrovascular disease mortality was associated with three day extreme events (99^th^percentile, lag0-2 (1.15: 0.98 – 1.34)) but not single day extreme events, nor was it associated with three day or single day severe events. We found all cardiovascular admissions were inversely associated with single day extreme and severe heat events lagged 1 day (95^th^percentile, lag1(0.96: 0.94 – 0.98) and these inverse associations were reflected in IHD (95^th^percentile, lag1(0.96:0.93 – 0.98)) and cerebrovascular disease (95^th^percentile, lag1(0.97: 0.88 – 0.97)).

Respiratory mortality was associated with single day and three-day severe events, but not with extreme events at (95^th^percentile, lag0 (1.14: 1.04 – 1.24). Pneumonia mortality was associated with single day extreme and severe events lag2 days (95^th^percentile, lag2 (1.25:1.05 – 1.49) but not three day severe or extreme events. COPD mortality was associated with single day severe events (95^th^percentile, lag0 (1.18:1.05 – 1.33), with a weaker association with three day severe events, while extreme events were not associated with COPD. Hospital admissions for all respiratory disorders were not associated with extreme events although there was a small magnitude association with three day and single day severe events (95^th^percentile, lag 0 (1.03:1.01 – 1.05)). While these results were reflected in pneumonia admissions and COPD admissions the associations were generally weak and inconsistent. These weak associations with respiratory outcomes were generally eliminated when air pollutants were included in the models, particularly ozone and nitrogen dioxide. We found in inverse association between childhood asthma admissions and single day severe and extreme events (95^th^percentile, lag0 (0.94: 0.89 – 1.01)). Controlling for air pollutants generally magnified this inverse association, particularly for ozone and nitrogen dioxide, such that three day severe and extreme events were also inversely associated with childhood asthma (heat + ozone model; 95^th^percentile, lag0-2 (0.91: 0.84 – 0.98)). Adult asthma admissions were inversely associated with both single day and three day extreme and severe events and controlling for air pollution generally give similar results (95^th^percentile, lag0 (0.92: 0.84 – 1.02)).

Mortality from renal failure was not associated with extreme or *severe* events. Three day and single day extreme and severe events were associated with hospital admissions for disorders of the renal system with the strongest single day associations at lag1 and lag2 days (95^th^percentile, lag1 (1.08: 1.05 – 1.12)). Controlling for air pollutants in the model reduced the magnitude and strength of these associations. Hospital admissions for urolithiasis (kidney stones) was associated with three day and single day extreme and severe events (95^th^percentile, lag1 (1.18:1.02 – 1.25)). Admissions for renal failure were associated with three day extreme and severe events as well as single day severe events but not extreme events (95^th^percentile, lag0 (1.26: 1.16 – 1.38)) although controlling for air pollutants reduced the magnitude of the associations with severe events and eliminated the association with three day extreme events.

Diabetes mortality was associated with single day severe events (95^th^percentile, lag0 (1.22:1.03 – 1.46) and weakly associated with three day severe events, but was not associated with extreme events. Hospital admissions for diabetes were associated with similar magnitude associations with three day and single day extreme and severe events (95^th^percentile, lag0 (1.12: 1.06 – 1.18)). Controlling for air pollutants generally gave similar magnitude associations for diabetes hospitalisations although the strength of these associations was reduced with three day extreme and severe events.

Deaths from all mental disorders were not associated with extreme or severe events for the all-ages group but were associated with single day extreme and severe events in the under 65 years age group lag 2 days (99^th^percentile, lag2 (2.84: 1.24 – 6.52) and 95^th^percentile, lag2 (1.53: 1.10 – 2.14)). Deaths from mental disorders (substance abuse) showed similar magnitude associations with single day and three day severe events (95^th^percentile, lag0 (1.38: 1.01 – 1.88)) as well as single day extreme events at lag1 and lag 2 days (99^th^percentile, lag1 (2.30: 1.19 – 4.44)) but not three day extreme events. Deaths from suicide were not associated with extreme events although there was a weak association with single day severe events lagged 3 days which only occurred in the under 65 year age group (95^th^percentile, lag3 (1.19: 0.98 – 1.44)). Mortality from dementia was inversely associated with single day severe and extreme events lagged 3 days, but not three day events (95^th^percentile, lag3, (0.83: 0.68 – 0.99)). Similarly, organic mental disorders were inversely associated with single day severe and extreme events lagged 3 days, but not three day events (95^th^percentile, lag3 (0.85: 0.71 – 1.02)).

Hospital admissions for all mental and behavioural disorders showed small magnitude associations with three day and single day extreme and severe events with the strongest single day association at lag1 days (95^th^percentile, lag1 (1.02:1.00 – 1.05) (Table 6). While these results were reflected in various categories of hospital admissions for mental and behavioural disorders the associations were generally weak and inconsistent. Hospital admissions for mood disorders were weakly associated with three day and single day severe events (95^th^percentile, lag1 (1.06:1.00 – 1.12)). Admissions for mental disorders related to substance abuse were weakly associated with three day and single day extreme events (99^th^percentile, lag0 (1.08: 0.97 – 1.20)). Admissions for dementia were associated with single day severe events (95^th^percentile, lag0 (1.14:0.99 – 1.31)) as were admissions for neurotic/ somatic/ stress (95^th^percentile, lag0, (1.06:0.99 – 1.14)). There was no consistent pattern of change in results for mental disorder admissions when air pollutants were included in the model with the magnitude of some associations decreasing, for example, dementia with ozone included in model (95^th^percentile, lag0 (1.08:0.92 – 1.28)). While the magnitude of some mental disorder admissions associations increased, for example, mental disorders related to substance abuse and PM2.5 (heat model: 99^th^percentile, lag0-2 (1.06: 0.96 – 1.18); heat + PM2.5 model: 99^th^percentile, lag0-2 (1.11: 0.99 – 1.25)). As hospital admissions for mental and behavioural disorders are unlikely to be causally related to air pollution, the change in the association with these outcomes suggests co-linearity may be influencing the ability of the model to assess the independent effects of heat and air pollution.

**Table 6:** Odds ratios of hospitalisations associated with severe and extreme heat (95th/99th percentile) in Sydney GMR

**Hospital admissions**		**Single day**		**3 day event**	
		**(Lag0)**		**(Lag0-2)**	
		**OR (95%CI)**		**(95%CI)**	
		**95th percentile**	**99th percentile**	**95th percentile**	**99th percentile**
All cause	All Age	*1.02 (1.01 - 1.03)*	*1.01 (1.00 - 1.02)*	*1.02 (1.01 - 1.03)*	*1.03 (1.02 - 1.05)*
	65+	*1.04 (1.03 - 1.06)*	*1.03 (1.01 - 1.06)*	*1.03 (1.02 - 1.04)*	*1.05 (1.03 - 1.07)*
All cardiovascular	All Age	1.01 (0.99 - 1.03)	*0.98 (0.94 - 1.03)*	0.98 (0.96 - 1.00)	0.98 (0.94 - 1.03)
	65+	1.01 (0.93 - 0.98)	0.98 (0.93 - 1.04)	0.97 (0.95 - 1.00)	0.96 (0.91 - 1.02)
IHD	All Age	1.04 (0.86 - 1.24)	*1.00 (0.93 - 1.06)*	0.99 (0.96 - 1.00)	1.00 (0.93 - 1.06)
	65+	*1.02 (0.98 - 1.06)*	1.02 (0.94 - 1.11)	*0.99 (0.95 - 1.03)*	0.99 (0.91 - 1.07)
All respiratory	All Age	1.03 (0.96 - 1.06)	0.98 (0.94 - 1.03)	*1.03 (1.01 - 1.05)*	1.01 (0.97 - 1.06)
	65+	*1.07 (1.04 - 1.10)*	1.03 (0.96 - 1.09)	*1.08 (1.04 - 1.11)*	*1.07 (1.01 - 1.14)*
Pneumonia	All Age	*1.04 (1.00 - 1.08)*	0.97 (0.89 - 1.06)	*1.05 (1.01 - 1.09)*	1.10 (1.01 - 1.20)
	65+	*1.06 (1.00 - 1.12)*	1.01 (0.90 - 1.13)	*1.09 (1.03 - 1.15)*	*1.12 (1.00 - 1.25)*
Asthma	1 - 14 yrs	*0.94 (0.89 - 1.01)*	*0.92 (0.80 - 1.04)*	*0.93 (0.87 - 0.98)*	0.89 (0.79 - 1.01)
	15 - 64 yrs	*0.96 (0.88 - 1.04)*	*0.86 (0.72 - 1.04)*	0.95 (0.87 - 1.04)	0.80 (0.66 - 0.97)
COPD	All Age	*1.06 (1.01 - 1.10)*	1.05 (0.97 - 1.14)	*1.06 (1.02 - 1.11)*	1.06 (0.97 - 1.15)
	65+	*1.07 (1.03 - 1.13)*	1.03 (0.94 - 1.14)	*1.07 (1.02 - 1.13)*	1.03 (0.93 - 1.14)
Diabetes	All Age	*1.12 (1.06 - 1.18)*	*1.16 (1.03 - 1.30)*	*1.07 (1.01 - 1.04)*	*1.14 (1.01 - 1.29)*
	65+	*1.12 (1.03 - 1.21)*	1.18 (0.99 - 1.39)	*1.06 (0.97 - 1.15)*	1.15 (0.95 - 1.37)
All renal disorders	All Age	*1.04 (1.01 - 1.10)*	*1.01 (0.94 - 1.08)*	*1.14 (1.04 - 1.11)*	*1.19 (1.12 - 1.27)*
	65+	*1.09 (1.04 - 1.14)*	1.01 (0.91 - 1.12)	*1.12 (1.07 - 1.17)*	*1.26 (1.15 - 1.40)*
Urolithiasis	All Age	1.01 (0.95 - 1.07)	1.05 (0.97 - 1.14)	*1.15 (1.08 - 1.22)*	*1.32 (1.18 - 1.50)*
	65+	1.07 (0.91 - 1.25)	0.90 (0.63 - 1.29)	*1.31 (1.12 - 1.53)*	*1.79 (1.34 - 2.40)*
Mental disorders	All Age	1.01 (0.98 - 1.04)	*1.08 (0.97 - 1.20)*	*1.03 (1.00 - 1.05)*	1.05 (0.99 - 1.10)
	65+	1.03 (0.96 - 1.09)	0.94 (0.81 - 1.09)	*1.09 (1.02 - 1.16)*	1.05 (0.91 - 1.20)
Dehydration	All Age	*1.78 (1.60 - 1.97)*	*2.16 (1.80 - 1.60)*	*1.72 (1.56 - 1.91)*	*2.32 (1.91 - 2.81)*
	65+	*1.85 (1.63 - 2.09)*	*2.08 (1.64 - 2.64)*	*1.78 (1.51 - 2.02)*	*2.15 (1.68 - 2.76)*
Heat related illness and injury	All Age	*12.05 (8.91 - 16.30)*	*18.76 (11.38 - 30.93)*	*1.77 (1.60 - 1.97)*	*10.35 (6.09 - 17.59)*
	65+	*19.47 (11.71 - 32.36)*	No data	*7.05 (4.69 - 10.60)*	No data
Disorders of fluid balance	All Age	*1.27 (1.14 - 1.42)*	*1.50 (1.20 - 1.86)*	*1.19 (1.06 - 1.33)*	*1.49 (1.19 - 1.86)*
	65+	*1.25 (1.10 - 1.42)*	*1.51 (1.18 - 1.93)*	*1.16 (1.02 - 1.32)*	*1.50 (1.16 - 1.94)*

Additional analyses that assessed the four alternative heatwave definitions designed to integrate the effect of high temperature over multiple days with all-cause, cardiovascular and respiratory mortality gave similar magnitude associations with these outcomes as the simpler heat wave definitions reported here.

## Discussion

We found that single day and three day severe heat events were associated with increased mortality and hospital admission in the Sydney GMR as well as less frequent and more intense extreme heat events. Our study found broadly similar results irrespective of the definition and metric (maximum temperature or apparent temperature) used to define heat events, consistent with the finding of Khalaj et al. and Barnett et al. [9,38]. While air pollution does not appear to confound the association between mortality and heat events, we found evidence of confounding by air pollution with some hospital admissions outcomes, particularly respiratory outcomes and ozone. The associations for some hospital admissions outcomes that were influenced by controlling for air pollutants in the statistical model are unlikely to be causally related to air pollution, for example heat related injury. This suggests that co-linearity between air pollutants and heat events may be influencing the ability of the model to assess the independent effects of heat and air pollution.

Our findings suggest mortality from disorders associated with fluid imbalance, as well as cardiovascular disease, pneumonia, COPD, diabetes, and mental disorders related to substance abuse are associated with heat events. As expected heat events were associated with substantial increases in hospital admissions for dehydration, other disorders of fluid, electrolyte and acid base balance, and heat related injury. We also found that heat events were associated with hospital admissions for renal disease, diabetes, and mental and behavioural disorders. These increases in mortality and hospitalisation during heat events place an additional load on already stretched emergency services, hospitals and the health system.

As expected hospital admission for heat related injury, disorders of fluid balance and dehydration had large magnitude associations with both single day and three day extreme and severe events. While deaths from dehydration were only weakly associated with severe events we found a large magnitude association with other fluid disorders and both severe and extreme events.

We found mortality and hospital admissions for mental disorders were associated with both single day and three day extreme and severe heat events. Deaths from all mental and behavioural disorders were associated with single day extreme and severe events in the under 65 year age group but not the all-age group. This was reflected in associations for hospital admissions for all mental and behavioural disorders with extreme and severe events. There was some consistency in the results for mortality and hospital admissions for mental disorders related to substance abuse with both outcomes associated with extreme events and mortality associated with severe events. The results for other sub categories of mortality and admissions for mental disorders were mixed. While we found inverse associations for mortality from organic mental disorders and dementia mainly with single day severe events these outcomes had very low daily counts with limited statistical power. We found weak positive associations with hospital admissions for mood, dementia, and somatic/neurotic/stress disorders and mainly single day severe events. Associations between heat events and mental and behavioural disorders have been found elsewhere [11,39]. A study in Adelaide, Australia found heat-waves were associated with mortality due to a range of mental health disorders [40]. The increased intensity and duration of heat-waves in Adelaide may explain the weaker associations with mortality we found in Sydney. Psychotropic drugs are commonly used by people with mental and behavioural disorders and these drugs may interfere with body temperature regulation to exacerbate the effects of heat-waves [39]. In Khalaj’s recent Sydney study, an association between hospital admission for mental and behavioural disorders, as both a primary diagnoses (compared with persons admitted for other conditions) and as an underlying diagnosis, (compared with persons admitted who did not have the condition) [9] was found. We found a weak association between suicide in the under 65 year age group and single day severe events. This result should be treated with caution as many papers have explored links between suicide and temperature and produced conflicting results [41-44]. Some studies have found a decreased suicide risk with increasing temperature [41], while in others an increased risk was observed [42]. One study found a U-shaped response with elevated suicide risk on extremely cold and warm days [43]. Our study contributes to the emerging literature in this area.

We found hospital admissions for renal failure, diabetes and urolithiasis were associated with extreme and severe heat events. While mortality from renal failure was not associated with heat events the small number of deaths coded to renal failure limit the statistical power of the analysis to find an association if one was present. A recent study in France found heat waves were associated with renal failure and that these effects were larger in elderly patients [45]. It is reasonable to assume that these associations may be related to increased fluid loss and subsequent altered fluid balance and dehydration.

While diabetes hospital admissions were associated with singe day and three day severe and extreme events, diabetes mortality was only weakly associated with single day and three day severe events. Once again the small number of deaths coded to diabetes limits the statistical power of the analysis to find an association if one were present. Disturbances to the fluid balance resulting from high temperature could also be a contributing factor to the observed associations with diabetes [46].

Cardiovascular disease mortality and the related outcomes of IHD and cerebrovascular disease were associated with single day and three severe and extreme events. Conversely we found weak inverse associations with single day extreme and severe events for all cardiovascular admissions, as well as IHD and cerebrovascular disease admissions. This suggests that heat events may lead to a rapid deterioration in existing cardiovascular disease resulting in death, and that this may occur during rare extreme events as well as the more frequent severe events. European studies have similarly found an association between heat-waves and cardiovascular mortality but not for hospital admissions where cardiovascular disease was the primary cause [47-51]. Khalaj’s 2010 Sydney case-only study did not find any association between heat-waves and hospital admissions for cardiovascular disease as the primary diagnosis (compared with persons admitted for other conditions) but did find an association with cardiovascular disease as an underlying diagnosis (compared with persons admitted who did not have the condition). This suggests that an underlying condition of cardiovascular disease modifies the effect of heat on hospital admissions for a range of other conditions [9,52].

Whilst respiratory mortality was associated with single day and three day severe events, it was generally not associated with extreme events, although once again the small number of deaths coded to respiratory mortality limits the statistical power of the analysis to find an association if one were present, especially for less frequent *extreme* events. While respiratory hospital admissions were associated with heat events all associations were eliminated when we controlled for the effects of air pollution, particularly ozone. Air pollution is associated with respiratory morbidity [53-55] and studies suggest that ozone can confound the association with heat for cardiovascular and respiratory outcomes [56]. Air pollution concentrations are influenced by weather and ozone in particular is correlated with temperature [57]. Heat and air pollution may have independent effects of respiratory outcomes however co-linearity may be influencing the ability of our statistical model to assess the independent effects of heat and air pollution and work is starting to emerge exploring the issue of confounding and effect modification of heat by air pollution [58]. More broadly, our finding that the effects of heat on some hospital admissions disease groups are sensitive to the effect of air pollution, particularly O_3_ and NO2, is consistent with previous work that identified this phenomenon in the 2003 heatwave in France [59].

We found an inverse association with single day and three day extreme events for childhood asthma, and for both severe and extreme events for adult asthma. Asthma time series data, particularly for children, is difficult to model [60]. Probably because children are more likely to experience frequent respiratory epidemics and because school holidays influence hospital admission rates [61]. The protective effect for asthma may also be due to the warm moist air associated with heat events in Sydney’s temperate climate. Asthma is known to by triggered by inhaling cold dry air, and in an experimental study, warm moist air has been shown to protect the airway disruption associated with exercise [62]. It is also possible that on very hot days, people with asthma stay inside more, and are reluctant to exercise due to concerns for triggering an asthma attack. A recent Sydney case-only study found heat waves were associated with increased hospital admissions with respiratory disease as an underlying diagnosis (compared with persons admitted who did not have the condition) suggesting that an underlying condition of respiratory disease modifies the effect of heat on hospital admissions for a range of other conditions [9].

Our sensitivity analyses suggest that, at least for mortality, the use of heatwave metrics designed to reflect multiple day events that meet detailed heatwave criteria produce broadly similar results to those found with single day heat metrics such as maximum daily air temperature, or a simple 3 day moving average.

The key strengths of this study include our use of long mortality and hospital admission time series, the wide range of diagnosis groups assessed our adjustment for the potential confounding effects of humidity and our assessment of the influence of air pollution on the heat associations. As with similar ecological time series studies we were limited by the lack of more detailed individual level data on exposure including other potential confounding factors such as the use of air conditioning during heat events, Similar to many other studies of the effects of heat on mortality or hospitalisation we used a time stratified case crossover analysis that has the benefit of controlling for the effects of unmeasured confounding factors that do not vary over time by design. An alternative statistical approach would be to use Poisson regression time series analysis. A recent paper by Guo et al. suggests that case crossover designs may not control for autocorrelation as effectively as time series analysis when assessing air pollution effects on mortality due in part to lack of control for seasonal effects. However, the generalizability of these concerns to our study assessing the effects of extreme heat in interrupted time series analyses of spring and summer months, and to our study location is unclear [63]. The different exposure metrics, different lag effects and large range of mortality and hospitalisation outcomes assessed in our study introduces the potential problem of multiple comparisons. This results in an increased probability of detecting a statistically significant finding by chance alone and the greater potential for spurious results to be reported. One approach to multiple testing suggests using more stringent P-values to account for the larger number of comparisons being conducted, although this can cause more problems than it intends to solve and no correction for multiple comparisons was made in our study [64]. Although this increased the chances of finding spurious associations, it reduced the chances of missing any important associations. Our results summary focuses on consistent lag effects rather than statistically significant effects with erratic lag structures.

This study highlights the increased risk of hospitalisation and death during heat events from cardiovascular disease, diabetes and mental health disorders. These chronic conditions are an important issue in today’s society [65]. The aged population are at increased risk of heat-related illness from a range of factors including decreased thermoregulation and an increased prevalence of chronic diseases disease, and these factors contribute to the increased risk of heat related death [66,67]. Many health agencies including the NSW Ministry of Health have implemented programs to improve management of chronic disease in the hope of reducing potentially avoidable admissions [68]. This study provides similar results to research conducted in the Northern Hemisphere regarding the effect of heatwaves on levels of mortality and morbidity and outlines the importance of including HHWS and heat management plans as part of chronic disease management.

## Conclusions

The purpose of this study was to provide data in the Australian context which will inform the development of a Heatwave Health Warning System for NSW. The findings of this study indicate that days with unusually high ambient temperatures increase mortality and hospitalisation from a range of conditions. Ebi and colleagues highlighted the costs and benefits of the establishment of a HHWS in Philadephia, with the benefits of implementing a HHWS far outweighing the costs [6]. We identified increases in mortality and morbidity for a range of conditions on both single and three day severe and extreme events, and a HHWS in NSW based on these thresholds would need to be activated on average one or two times per year for extreme events and around ten times per year for more frequent severe events. Compliance with a HHWS is a critical factor in maximising the protective effect of the warning system and frequent alerts may reduce compliance in the target population. It is recommended that in NSW the HHWS be implemented at the first one or two occurrences of temperatures above the 95th percentile of average monthly temperature (severe event level) from the beginning of spring (September) and thereafter at all occurrences of temperatures above the 99th percentile of average monthly temperature (extreme event level) until the end of summer (February) in order to maximise health protection and reduce complacency. Public health messages associated with all heat health warnings should emphasise the importance of maintaining hydration, particularly for people who have chronic diseases whether the temperature be severe or extreme.

## Abbreviations

BoM: Bureau of meteorology; COPD: Chronic obstructive pulmonary disease; GMR: Greater metropolitan region; HHWS: Heatwave health warning system; ICD – 10: International classification of diseases (Version 10); LGA: Local government area; NSW: New South Wales, Australia; SLA: Statistical local area; TMax: Maximum daily temperature; T(App)Max: Apparent maximum daily temperature.

## Competing interests

The authors declare that they have no competing interests.

## Authors’ contributions

GM, FJ and BJ conceived and developed the overall study and conducted the data reviews. LW helped develop the study, assisted with data analysis and prepared the manuscript. IH developed the analysis methods and conducted data analysis. HA-Y conducted data analysis. RB and CG critically reviewed the manuscript and provided advice during study development. All authors read and approved the final manuscript.

## Supplementary Material

Additional file 1 ICD-10 Codes used in analysis. Click here for file

## References

[B1] Intergovernmental Panel on Climate Change (IPCC) Solomon SD, Qin M, Manning Z, Chen M, Marquis KB, Avery M, Tignor , Miller HL Summary for Policymakers Climate Change 2007: The Physical Science Basis. Contribution of Working Group I to the Fourth Assessment Report of the Intergovernmental Panel on Climate Change 2007 5 ipcc.ch/pdf/assessment-report/ar4/wg1/ar4-wg1-spm.pdf

[B2] Kovats RS Hajat S Heat stress and public health: a critical review Annu Rev Public Health 2008 29 41 55 10.1146/annurev.publhealth.29.020907.090843 18031221

[B3] Kilbourne EM Heat-related illness: current status of prevention efforts Am J Prev Med 2002 22 328 329 10.1016/S0749-3797(02)00412-9 11988389

[B4] Bernard SM McGeehin MA Municipal heat wave response plans Am J Public Health 2004 94 1520 1522 10.2105/AJPH.94.9.1520 15333307 PMC1448486

[B5] Nogueira PJ Kirch WMB, Bertollini R Examples of heat health warning systems: Lisbon’s ICARO surveillance system, summer of 2003 Extreme Weather Events and Public Health Responses 2005 Darmstadt, Germany: Springer-Verlag 141 160

[B6] Ebi K Tiesberg TJ Kalkstein LS Robinson L Weiher RF Heat watch/warning systems save lives: estimated costs and benefits for Philadelphia 1995 – 1998 Am Meteorol Soc 2004 1067 1073

[B7] Sheridan SC Kalkstein LS Progress in heat watch-warning system technology Bull Am Meteorol Soc 2004 85 1931 1941 10.1175/BAMS-85-12-1931

[B8] Anderson BG Bell ML Weather-related mortality: How heat, cold and heat waves affect mortality in the United States Epidemiology 2009 20 205 213 10.1097/EDE.0b013e318190ee08 19194300 PMC3366558

[B9] Khalaj B Lloyd G Sheppeard V Dear K The health impacts of heatwaves in five regions of New South Wales, Australia: a case-only analysis Int Arch Environ Health 2010 83 833 842 10.1007/s00420-010-0534-2 20464412

[B10] Garssen J Harmsen C de Beer J The effect of the summer 2003 heat wave on mortality in the Netherlands Eurosurveillance 2005 10 pii-577 16088044

[B11] Johnson H Kovats RS McGregor G Stedman J Gibbs M Walton H The impact of the 2003 heat wave on mortality and hospital admissions in England Health Stat Q 2005 25 6 11 15804164

[B12] Fouillet A Rey G Laurent F Pavillion G Bellec S Guihenneuc-Jouyaux C Clavel J Jougla E Hemon D Excess mortality related to the august 2003 heat wave in France Int Arch Occup Environ Health 2006 80 16 24 10.1007/s00420-006-0089-4 16523319 PMC1950160

[B13] Knowlton K Rotkin-Ellman M King G Margolis HG Smith D Solomon G The 2006 California heat wave: impacts on hospitalisations and emergency department visits Environ Health Perspect 2009 94 1520 1522 10.1289/ehp.11594 PMC2627866 19165388

[B14] Hayhoe K Sheridan S Kalkstein L Greene S Climate change, heat waves, and mortality projections for Chicago J Great Lakes Res 2010 36 65 73

[B15] Kalkstein L Sheridan S The social impacts of the heat–health watch/warning system in Phoenix, Arizona: assessing the perceived risk and response of the public Int J Biometeorol 2007 52 43 55 doi:10.1007/s00484-006-0073-4 10.1007/s00484-006-0073-4 17262221

[B16] Guest CS Willson K Woodward AJ Climate and mortality in Australia: retrospective study, 1979–1990, and predicted impacts in five major cities in 2030 Clim Res 1999 13 1 15

[B17] McMichael AJ Human health and climate change in oceania: a risk assessment Commonwealth Department of Ageing 2003 Canberra, ACT. Australia: Commonwealth Department of Ageing

[B18] Nitschke M Tucker GR Bi P Morbidity and mortality during heatwaves in metropolitan Adelaide Med J Aust 2007 187 662 665 18072911 10.5694/j.1326-5377.2007.tb01466.x

[B19] Bi P Parton KA Wang J Donald K Temperature and direct effects on population health in Brisbane, 1986–1995 J Environ Health 2008 70 48 53 18468224

[B20] Vaneckova P Beggs PJ Jacobson CR Spatial analysis of heat-related mortality among the elderly between 1993 and 2004 in Sydney, Australia Soc Sci Med 2010 70 293 304 10.1016/j.socscimed.2009.09.058 19880232

[B21] Hansen AL Bi P Ryan P Nitschke M Pisaniello D Tucker G The effects of heat waves on hospital admissions for renal disease in a temperate city of Australia Int J Epidemiol 2008 37 1359 1366 10.1093/ije/dyn165 18710886

[B22] Australian Bureau of Meteorology 2012 http://reg.bom.gov.au/announcements/media_releases/nsw/2004febnsw.shtm

[B23] Australian Bureau of Statistics Causes of Death Australia (3303.0). Australian Bureau of Statistics 2011 Commonwealth of Australia

[B24] NSW Department of Health Inpatient Statistics Collection (ISC) 2010 Sydney Australia: NSW Government

[B25] Wilmore A Population Weighted Concordance Between ASGC Versions Between 1998 and 2005 with ASGC 2006, NSW Health Department, 2006 2006 Sydney Australia: NSW Government

[B26] National Climate Centre of the Bureau of Meteorology National Climate Centre of the Bureau of Meteorology: Daily or three hourly weather data for Bureau of Meteorology stations 2010 Australia: Government of Victoria

[B27] Dear K Ranmuthugala G Kjellström T Skinner C Hanigan I Effects of temperature and ozone on daily mortality during the August 2003 heat wave in France Arch Env Occup Health 2005 60 205 212 10.3200/AEOH.60.4.205-212 17214291

[B28] Australian Bureau of Statistics, 2007 Australian Bureau of Statistics. Australian Census 2006 Basic Community Profiles for Census Collector’s Districts Data Pack (Cat No. 2069.0.30.001 Second Release) 2007 Canberra, Australia: Commonwealth of Australia

[B29] Nicholls N Skinner C Loughnan M Tapper N Temperature Thresholds Associated with Increased Mortality in ten Major Population Centres in Rural Victoria 2009 Monash University: Melbourne

[B30] Steadman RG The assessment of sultriness: part I: a temperature-humidity index based on human physiology and clothing science J Appl Meteorol 1979 18 861 873 10.1175/1520-0450(1979)018<0861:TAOSPI>2.0.CO;2

[B31] Steadman RG The assessment of sultriness: part II: effect of wind, extra radiation. And barometric pressure on apparent temperature J Appl Meteorol 1979 18 874 884 10.1175/1520-0450(1979)018<0874:TAOSPI>2.0.CO;2

[B32] Kalkstein LS Valimont KM An evaluation of summer discomfort in the United States using a relative climatological index Bulletin of the American Meteorological Society 1986 7 842 848

[B33] Steadman RG Norms of apparent temperature in Australia Aust Meteorol Mag 1994 43 http://www.bom.gov.au/amm/docs/1994/steadman.pdf

[B34] Nairn J Fawcett R Ray D Defining and Predicting Excessive Heat Events, a National System. Centre for Australian Weather and Climate Research Modelling Workshop 2009 2009 Melbourne: Modelling & Understanding High Impact Weather http://www.cawcr.gov.au/events/modelling_workshops/workshop_2009/papers/NAIRN.pdf

[B35] Broome R NSW Ministry of Health (pers communication) 2011 NSW Health

[B36] Jaakkola JJ Case-crossover design in air pollution epidemiology Eur Respir J Suppl 2003 40 81s 5s 12762580 10.1183/09031936.03.00402703

[B37] Janes H Sheppard L Lumley T Case-crossover analyses of air pollution exposure data: referent selection strategies and their implications for bias Epidemiology 2005 16 717 26 10.1097/01.ede.0000181315.18836.9d 16222160

[B38] Barnett AG Tong S Clements AC What measure of temperature is the best predictor of mortality? Environ Res 2010 110 604 11 10.1016/j.envres.2010.05.006 20519131

[B39] García-Pina R Tobías Garcés A Sanz Navarro J Navarro Sánchez C García-Fulgueiras A Effect of weather temperature on hospital emergencies in the Region of Murcia, Spain, throughout the 2000–2005 and its use in epidemiological surveillance Rev Esp Salud Publica 2008 82 153 66 10.1590/S1135-57272008000200002 18496620

[B40] Hansen A Bi P Nitschke M Ryan P Pisaniello D Tucker D The effects of heatwaves on mental health in a temperate Australian city Environ Health Perspect 2008 116 1369 1375 10.1289/ehp.11339 18941580 PMC2569097

[B41] Dixon P Kalkstein A Climate-suicide relationships: a research problem in need of geographic methods and cross-disciplinary perspectives Geography Compass 2009 3 1 14 10.1111/j.1749-8198.2008.00200.x

[B42] Tsai J-F Socioeconomic factors outweigh climate in the regional difference of suicide death rate in Taiwan Psychiatry Res 2010 179 212 6 10.1016/j.psychres.2008.06.044 20483166

[B43] Kim Y Kim H Kim D-S Association between daily environmental temperature and suicide mortality in Korea (2001–2005) Psychiatry Res 2011 186 390 6 10.1016/j.psychres.2010.08.006 20828832

[B44] Wang Y Wang D Wang X Suicide and meteorological factors in Huhhot, Inner Mongolia Crisis: The Journal of Crisis Intervention and Suicide Prevention 1997 18 115 7 10.1027/0227-5910.18.3.115 9454999

[B45] Josseran L Caillère N Brun-Ney D Rottner J Filleul L Brucker G Astagneau P Syndromic surveillance and heat wave morbidity: a pilot study based on emergency departments in France BMC Med Inform Decis Mak 2009 9 14 doi:10.1186/1472-6947-9-14 10.1186/1472-6947-9-14 19232122 PMC2654446

[B46] Timiras P Physiological Basis of Ageing and Geriatrics 2007 4 CRC Press

[B47] Hausfater P Doumenc B Chopin S Le Manach Y Santin A Dautheville S Patzak A Hericord P Mégarbane B Andronikof M Terbaoui N Riou B Elevation of cardiac troponin I during non-exertional heat-related illnesses in the context of a heatwave Crit Care 2010 14 R99 10.1186/cc9034 20507603 PMC2911736

[B48] Mastrangelo G Hajat S Fadda E Buja A Fedeli U Spolaore P Contrasting patterns of hospital admissions and mortality during heat waves: are deaths from circulatory disease a real excess or an artifact? Med Hypotheses 2006 66 1025 1028 10.1016/j.mehy.2005.09.053 16413137

[B49] Diaz J Linares C Impact of extreme temperatures on daily mortality in Madrid (Spain) among the 45–64 age-group Int J Biometeorol 2006 50 342 348 doi:10.1007/s00484-006-0033-z 10.1007/s00484-006-0033-z 16718468

[B50] Kovats RS Hajat S Wilkinson P Contrasting patterns of mortality and hospital admissions during hot weather and heat waves in Greater London, UK Occup Environ Med 2004 61 898 898 10.1136/oem.2003.012047 PMC1757853 15477282

[B51] Morgan G Sheppeard V Khalaj B Ayyar A Lincoln D Jalaludin B The effects of bushfire smoke on daily mortality and hospital admissions in Sydney, Australia, 1994 to 2002 Epidemiology 2010 21 47 55 10.1097/EDE.0b013e3181c15d5a 19907335

[B52] Wang XY Barnett AG Yu W FitzGerald G Tippett V Aitken P Neville G McRae D Verrall K Tong S The impact of heatwaves on mortality and emergency hospital admissions from non-external causes in Brisbane 2010 Occ Env Med Online: Australia doi:10.1136/oem.2010.062141 10.1136/oem.2010.062141 21719563

[B53] Simpson R Williams G Petroeschevsky A The short-term effects of air pollution on hospital admissions in four Australian cities Aust NZ J Public Health 2005 29 213 221 15991768

[B54] Simpson R Williams G Petroeschevsky A The short-term effects of air pollution on mortality in four Australian cities Aust N Z J Public Health 2005 29 205 212 10.1111/j.1467-842X.2005.tb00758.x 15991767

[B55] Knowlton K Rosenthal JE Hogrefe C Lynn B Gaffin S Goldberg R Assessing ozone-related health impacts under a changing climate Environ Health Perspect 2004 112 1557 1563 10.1289/ehp.7163 15531442 PMC1247621

[B56] Derwent RG Holgate S, Samet J, Koren H Atmospheric chemistry Air Pollution and Health 1999 New York: Academic Press

[B57] Ren C Williams GM Morawska L Mengersen K Tong S Ozone modifies associations between temperature and cardiovascular mortality: analysis of the NMMAPS data Occup Environ Med 2008 65 255 260 10.1136/oem.2007.033878 17890300

[B58] Schwartz J Spix C Touloumi G Methodological issues in studies of air pollution and daily counts of deaths or hospital admissions J Epidemiol Community Health 1996 50 S3 S11 10.1136/jech.50.Suppl_1.S3 8758217 PMC1060881

[B59] Filleul L Cassadou S Médina S Fabres P Lefranc A Eilstein D Le Tertre A Pascal L Chardon B Blanchard M The relation between temperature, ozone, and mortality in nine French cities during the heat wave of 2003 Environ Health Perspect 2006 114 1344 1347 10.1289/ehp.8328 16966086 PMC1570046

[B60] Storr J Lenney W School holidays and admissions with asthma Arch Dis Child 1989 64 103 107 10.1136/adc.64.1.103 2923458 PMC1791812

[B61] Lincoln D Morgan G Sheppeard V Jalaludin B Corbett S Beard J Return to school after term holidays is associated with increased hospital admissions for childhood asthma in Sydney, Australia Public Health 2006 120 854 862 10.1016/j.puhe.2006.05.015 16904142

[B62] Bolger C Tufvesson E Anderson SD The effect of inspired air conditions on exercise-induced bronchoconstriction and urinary CC16 levels in athletes J Appl Physiol 2011 111 1059 1065 10.1152/japplphysiol.00113.2011 21799131

[B63] Guo Y Barnett AG Zhang Y Tong S Yu W Pan X The short-term effect of air pollution on cardiovascular mortality in Tianjin, China: comparison of time series and case–crossover analyses Sci Total Environ 2010 2010 409 300 306 21055792 10.1016/j.scitotenv.2010.10.013

[B64] Rothman KJ No adjustments are needed for multiple comparisons Epidemiology 1990 1 43 46 10.1097/00001648-199001000-00010 2081237

[B65] Australian Institute of Health and Welfare Ageing and aged care in Australia July 2008 2008 Canberra, Australia: Commonwealth of Australia 1 45

[B66] Wilson L Black DA Veitch C Heatwaves and the elderly: the role of the GP in reducing morbidity Aust Fam Physician 2011 40 637 40 21814665

[B67] Astrom DO Forsberg B Rocklov J Heat wave impact on morbidity and mortality in the elderly population: a review of recent studies Maturitas 2011 69 99 105 10.1016/j.maturitas.2011.03.008 21477954

[B68] NSW Ministry of Health (2012) Chronic care management program 2012 http://www0.health.nsw.gov.au/cdm/severe_chronic_disease_management_program.asp

