# Supplementary material for: The impact of heat on mortality and morbidity in the Greater Metropolitan Sydney Region: a case crossover analysis
Source: Environ Health. 2013 Nov 15;12:98. doi: 10.1186/1476-069X-12-98 (PMC3842658; doi:10.1186/1476-069X-12-98)
Supplement: Additional file 1 — ICD-10 Codes used in analysis. [file 1476-069X-12-98-S1.docx]

**Additional file 1: ICD-10 Codes used in analysis**

| **ICD CODES ANALYSED** | **Mortality** | **Hospitalisations** |
| --- | --- | --- |
| A00 - R99 All non-external causes |  |  |
| I00 – I99 All diseases of the circulatory system |  |  |
| I60 – I69 Cerebrovascular diseases |  |  |
| I20 – I25 Ischaemic heart disease |  |  |
| J00 – J99 All diseases of the respiratory system |  |  |
| J12 – J18 Pneumonia |  |  |
| J45 – J46 Asthma | Not analysed |  |
| J40 – J44, J47 Chronic obstructive pulmonary disease (COPD) |  |  |
| N00 – N39 All diseases of the renal system | Not analysed |  |
| N17 – N19 Renal failure |  |  |
| N20 – N23 Urolithiasis | Not analysed |  |
| F00 – F03 Dementia |  |  |
| F00-F99 Mental and behavioural disorders |  |  |
| F10 – F19 Mental disorders associated with substance abuse |  |  |
| F20 – F29 Schizophrenia and schizotypal disorders | Not analysed |  |
| F30 – F39 Mood disorders | Not analysed |  |
| F40 – F48 Neurotic / somatic / stress disorders | Not analysed |  |
| E10-E14 Diabetes mellitis |  |  |
| F00-F09 Organic mental disorders |  |  |
| X60 – X84 or Y87 Suicide |  | Not analysed |
| S00 – T98 All injury | Not analysed |  |
| T67 Heat related injury | Not analysed |  |
| E86 Dehydration |  |  |
| E87 – E87.8 Other disorders of fluid,  Electrolyte and acid balance |  |  |
